# Anti-nuclear autoantibodies in Graves’ disease and Graves’ orbitopathy

**DOI:** 10.1007/s40618-022-01906-3

**Published:** 2022-08-27

**Authors:** G. Lanzolla, L. Puccinelli, M. Giudetti, S. Comi, F. Menconi, M. N. Maglionico, C. Posarelli, M. Figus, C. Marcocci, M. Marinò

**Affiliations:** 1 Department of Clinical and Experimental Medicine, Endocrinology Unit I, University of Pisa and University Hospital of Pisa, Via Paradisa 2, 56124 Pisa, Italy; 2 Department of Surgical, Medical and Molecular Pathology, Ophthalmology Unit I, University of Pisa and University Hospital of Pisa, Via Paradisa 2, 56124 Pisa, Italy

**Keywords:** Graves’ disease, Graves’ orbitopathy, Graves’ ophthalmopathy, Thyroid eye disease, Anti-nuclear autoantibodies, Non-organ-specific autoantibodies

## Abstract

**Objective:**

A relationship between thyroid and non-organ-specific autoimmunity could be relevant for Graves’ orbitopathy (GO), which affects connective tissue. We investigated the association between GO and anti-nuclear antibodies (ANAs).

**Methods:**

Retrospective investigation was conducted in 265 patients with Graves’ disease (GD), 158 with and 107 without GO. Primary outcome was: prevalence of ANAs in GO vs no-GO. Secondary outcomes were: (1) relationship between ANAs and GO features; (2) prevalence of ANAs in GD compared with non-autoimmune hyperthyroidism [(78 patients with toxic nodular goiter (TNG)]; (3) distribution of ANA patterns.

**Results:**

ANAs were detected in 212 (80%) GD patients, but prevalence did not differ between GO (79.7%) and no-GO (80.3%). Higher ANA titers (1:160) were more common in GO (51.5 vs 38.3%), but only nearly significantly (OR 0.5; 95% CI: 0.3–1; *P* = 0.059). Proptosis was lower in ANA-positive patients (mean difference: − 1.4 mm; 95% CI from − 2.5 to − 0.3; *P* = 0.011), in whom nearly significantly lower CAS (Mann–Whitney *U*: 1.5; *P* = 0.077) and eyelid aperture (mean difference: − 0.9 mm; 95% CI from − 2 to 0; *P* = 0.062) were observed. Prevalence of ANAs in GD was lower than in TNG (80 vs 91%; OR 0.3; 95% CI: 0.1–0.9; *P* = 0.028), but nuclear speckled pattern was more frequent (OR 22.9; 95% CI 1.3–381.3; *P* = 0.028).

**Conclusions:**

Although ANAs are not more frequent in GO, they seem to exert a protective role on its severity and on development of GD. A switch of T cell population in ANA-positive patients, resulting in a different phenotype, may be responsible. Further studies are needed to investigate the mechanisms.

## Introduction

Non-organ-specific autoantibodies are generally found in patients suffering from connective tissue disorders, but they can also be observed in patients with organ-specific autoimmunity or in the otherwise healthy subjects, with a prevalence that increases with age [1, 2]. They are generally considered poorly specific and their titer is not necessarily correlated with the presence of a disease or with the severity of the clinical picture when disease is present. Non-organ-specific autoantibodies are not pathogenetic per se, but are rather considered an epiphenomenon within the picture of non-organ-specific autoimmunity [1, 2].

Within non-organ-specific antibodies, the most common are anti-nuclear antibodies (ANAs) [1–4]. ANAs are generally measured by indirect immunofluorescence and the staining pattern reflects ANA subtypes and localization of the antigen [5, 6]. In the fields of rheumatology and immunology, the study of ANA patterns is a topic of great interest, as different patterns may have different clinical, diagnostic and prognostic implications. For this purpose, the International Consensus on Antinuclear Antibody (ANA) Patterns (ICAP) was created in 2015. The most frequently observed staining patterns are nuclear, with numerous subtypes, being the dense fine speckled (AC-02 according to the ICAP nomenclature) the one most commonly observed in healthy subjects [5–7].

An association between autoimmune thyroid diseases and rheumatological disorders has been reported since a long time [8, 9]. The most common non-organ-specific diseases associated with thyroid autoimmunity are Sjögren’s syndrome, systemic lupus erythematosus and rheumatoid arthritis. Patients with systemic rheumatological diseases may have antibodies to thyroglobulin or thyroperoxidase, with a prevalence ranging from ~ 10 to ~ 30% [9]. Based on the hypothesis that production of ANAs may represent the consequence of disruption of Th1/Th2 balance as well as of Th17/Treg balance, the presence of detectable ANAs in patients with autoimmune thyroid diseases has also been investigated, although data are somehow conflicting. Furthermore, to our knowledge, the frequency of ANAs in patients with Graves’ orbitopathy (GO) is virtually unknown, which could be relevant considering that GO affects orbital connective tissue [10–16]. Therefore, we conducted a retrospective investigation aimed at assessing the prevalence of ANAs in patients with GO, compared with patients with Graves’ disease (GD), but without GO. In addition, we evaluated the relationship between ANAs and GO features and the prevalence of ANAs in GD compared with non-autoimmune hyperthyroidism, namely toxic nodular goiter (TNG).

## Subjects and methods

### Study design

The study was aimed at investigating retrospectively the association between GO and ANAs, and entailed the analysis of data of all consecutive patients with GD who came to our observation to undergo radioiodine treatment over a period of 30 consecutive months.

### Setting

The study was performed in a tertiary referral center, namely the University Hospital of Pisa. Patients were included by consecutive sampling. Inclusion and exclusion criteria are reported below . Patient data were recorded in a database. The following database validation procedures were used: allowed character checks, batch totals, missing records check, cardinality check, digits check, consistency check, control totals, cross-system consistency check, data type check, hash totals, limit check, logic check, presence check, range check, spelling and grammar check, and uniqueness check.

### Participants

Inclusion criteria were: (1) male and female patients aged 18–85 years; (2) a diagnosis of GD, based on hyperthyroidism associated with detectable circulating anti-TSH-receptor autoantibodies (TRAbs); (3) informed consent to data use.

Exclusion criteria were: (1) treatment with glucocorticoids (GC) or any immunosuppressive medication in the preceding 12 weeks; (2) absence of informed consent.

A total of 265 GD patients satisfied the inclusion criteria and evaded the exclusion criteria and were, therefore, studied. In addition, data from 78 consecutive patients with toxic nodular goiter (TNG) who came at our observation over the same period of time were used as a non-autoimmune control.

The study was performed according to Institutional guidelines and with the International Conference on Harmonization Good Clinical Practice guidelines and the principles of the Declaration of Helsinki.

### Outcomes

The primary outcome of the study was the prevalence of detectable ANAs in GD patients based on the presence or absence of GO. The diagnosis of GO was based on the presence of at least one of the following eye features: (1) proptosis ≥ 2 mm compared with normal values for sex and race; (2) presence of diplopia; (3) lid retraction ≥ 2 mm; and (4) a clinical activity score (CAS) ≥ 2 out of 7 points. The secondary outcomes were: (1) relationship between ANAs and GO features; (2) prevalence of ANAs in GD compared with TNG; (3) distribution of ANA florescence patterns.

### Sources of data and measurements

An ophthalmological evaluation had been performed in all patients, including: (1) exophthalmometry; (2) measurement of eyelid aperture; (3) evaluation of CAS; (4) assessment of diplopia; (5) assessment of the corneal status; (6) examination of the fundi; and (7) measurement of visual acuity. The following serum tests had been performed in all subjects prior to radioiodine treatment: (1) FT4 and FT3 (Vitros Immunodiagnostics, Raritan, NJ); (2) TSH (Immulite 2000, Siemens Healthcare, Gwynedd, UK); (3) anti-TSH receptor autoantibodies (TRAbs) (Brahms, Berlin, Germany); (4) ANAs, by immunofluorescence (Euroimmun, Lübeck, Germany). All patients underwent a thyroid ultrasound with measurement of thyroid volume. Thyroid ultrasound was performed using a real time instrument (Esaote SPA, Genova, Italy; My Lab 50 machine with 7.5–12 MHz linear transducer). The volume of thyroid lobes was calculated according to the ellipsoid formula.

### Statistical analyses

Continuous variables are presented as mean (SD) or median (IQR). The following tests were performed: (1) ANOVA with Bonferroni’s correction; (2) Mann–Whitney; (3) two-tailed Fisher’s exact test; and (4) Chi-square.

## Results

### Participants

As shown in Table 1, data from 265 patients with GD who came to our observation between February 1st 2019 and August 1st 2021 were analyzed. One-hundred and fifty eight of them (59.6%) had a clinical evidence of GO. This figure is greater than the ones reported by the most recent literature [6], which probably reflects the fact that being a tertiary referral center, a high proportion of GO patients are seen. All patients came to our observation for radioiodine treatment of hyperthyroidism and had, therefore, withdrawn anti-thyroid medications 3–5 days before data collection. There was no difference between patients with GO and those without GO concerning gender, smoking habits, TSH, FT3, FT4, TRAbs and ultrasound thyroid volume. As expected from previous epidemiological observations [17], GO patients were slightly older than those without GO. In addition, the duration of hyperthyroidism was significantly shorter in GO patients, reflecting a more aggressive approach in our Center in terms of definitive treatment of hyperthyroidism when GO is present [18].

**Table 1:** Demographic and clinical features of patients with Graves’ disease based on the presence or absence of Graves’ orbitopathy (GO)

	GO (*n* = 158)	No GO (*n* = 107)	Statistics
Gender	Males: 35 (22.1) Females: 123 (77.8)	Males: 21 (19.6) Females: 86 (80.3)	OR: 1.6 95% CI from 0.6 to 2.1 *P* = 0.62
Age (years)	49.7 (13.3)	42.5 (14.1)	Mean difference: − 7.1 95% CI from − 10.5 to − 3.7 *P* < 0.0001
Smoking	Never smokers: 75 (47.4) Ex-smokers: 23 (14.5) Current smokers: 56 (35.4)	Never smokers: 63 (58.8) Ex-smokers: 10 (9.3) Current smokers: 30 (28)	Chi^2^: 4 *P* = 0.13
Time since diagnosis of hyperthyroidism (months)	24 (12–39)	40 (24–84)	Mann–Whitney *U*: 4.9 *P* < 0.0001
TSH (mU/L) NV: 0.4–4	0.5 (0–1.6)	0.4 (0–1.2)	Mann–Whitney *U*: 7.5 *P* = 0.12
FT3 (ng/L) NV: 2.7–5.7	4.3 (3.4–5.4)	4.6 (3.9–6)	Mann–Whitney *U*: 7.3 *P* = 0.061
FT4 (ng/dL) V.N: 0.70–1.70	1.1 (0.9–1.5)	1.1 (0.9–1.3)	Mann–Whitney *U*: 7.9 *P* = 0.44
TRAbs (UI/L) NV: < 1.5	3 (1.6–7.9)	2.8 (1.2–7.2)	Mann–Whitney *U*: 7.7 *P* = 0.24
Thyroid volume (mL)	15.5 (12.1–22.4)	16.3 (11.7–22.1)	Mann–Whitney *U*: 7.9 *P* = 0.69

Data are *n* (%), mean (SD) or median (IQR)

*NV* normal values, *TRAbs* anti-TSH receptor autoantibodies

As shown in Table 2, where the eye features of the 158 GO patients are reported, the majority of them had a mild, inactive GO, according to the criteria proposed by the European Group on Graves’ Orbitopathy (EUGOGO) [10].

**Table 2:** Eye features in 158 patients with Graves’ orbitopathy (GO)

Parameter	
Proptosis (mm)	20.6 (2.7)
Clinical activity score	2 (1–3)
Eyelid aperture (mm)	11.8 (2.4)
Diplopia	Absent: 113 (71.5) Intermittent: 18 (11.3) Inconstant: 16 (10.1) Constant 11 (6.9)
Visual acuity (decimals)	0.98 (0.09)
GO duration (mo.)	15 (9–36)
GO degree	Mild: 115 (72.7) Moderate-to-severe: 43 (27.2) Severe: 0 (0)

Data are mean (SD), median (IQR), or *n* (%)

### Prevalence of ANAs in the study population

Of the 265 patients studied, 212 (80%) had detectable ANAs, all of them at low titers, namely between 1:80 (98 patients, 46.2%) and 1:160 (114 patients, 53.7%), whereas ANAs were not detected at greater dilutions. The presence of detectable ANAs was not significantly affected by gender (females vs males, OR 1.8, 95% CI from 0.9 to 3.6), although, as expected [1–4], it was slightly greater in females (172/209, 82.2%) than in males (40/56, 71.4%). In addition, the presence of ANAs was not affected by age, which was 45.8 year. (12.3) in ANA-negative and 47.1 years. (14.5) in ANA-positive patients (mean difference: − 1.2 years, 95% CI from − 5.5 to 9.9). Duration of hyperthyroidism and smoking did not affect the presence of detectable ANAs (not shown).

### Primary outcome: ANAs and GO

**Fig. 1:**
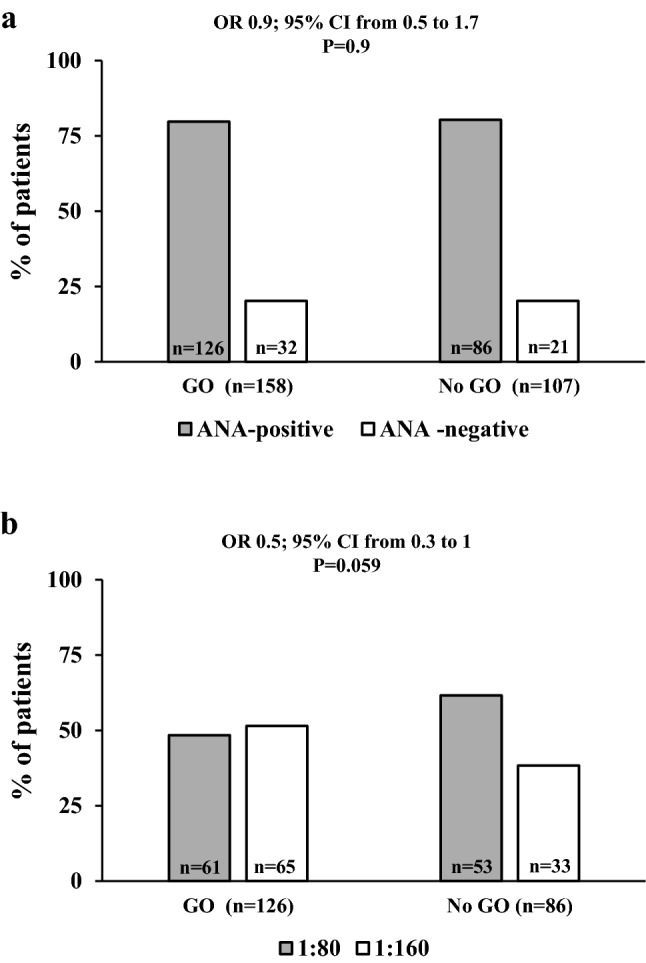
Anti-nuclear antibodies (ANAs) in patients with Graves’ disease. **a** Prevalence of ANAs according to the presence of Graves’ orbitopathy (GO); **b** distribution of ANA titers in ANA-positive patients according to the presence of GO

As shown in Fig. 1a, the prevalence of detectable ANAs did not differ between patients with GO and those without GO. However, the prevalence of higher ANA titers (1:160) was greater in GO patients, although the difference did not reach statistical significance (Fig. 1b). The distribution of ANA patterns did not differ between patients with or without GO (not shown).

### Secondary outcome-1: relationship between ANAs and GO features

**Fig. 2:**
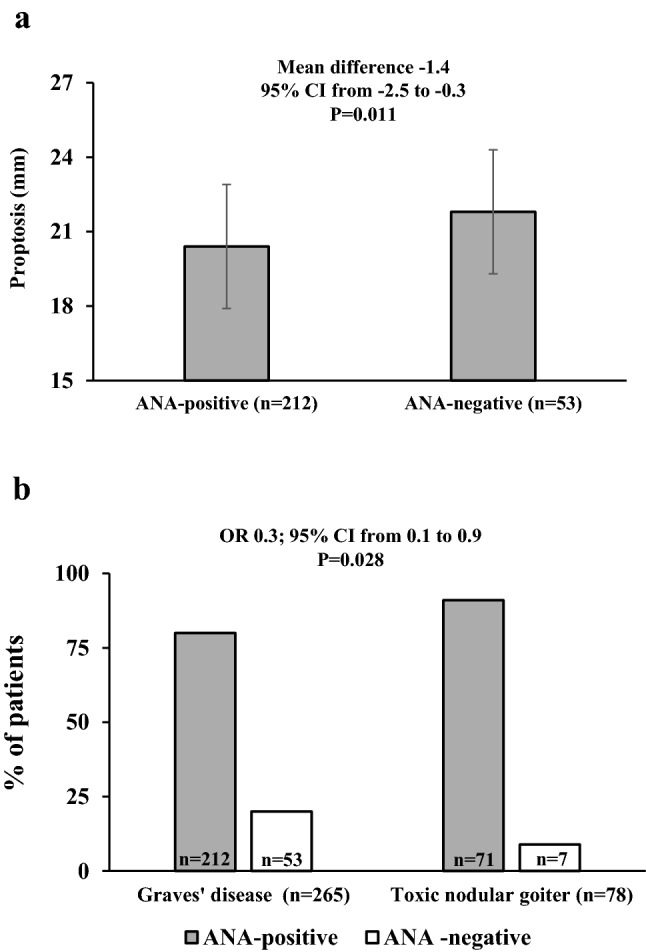
**a** Proptosis in patients with Graves’ orbitopathy (GO), based on the presence of detectable anti-nuclear antibodies (ANAs). **b** Prevalence of anti-nuclear antibodies (ANAs) in patients with Graves’ disease vs toxic nodular goiter

As shown in Fig. 2a, within GO patients, the extent of proptosis was significantly lower in ANA-positive patients. In addition, CAS was lower in ANA-positive patients (Mann–Whitney *U*: 1.5; *P* = 0.077), who had a lower prevalence of patients with active GO (CAS ≥ 3 points) (34.9 vs 53.1%; OR 0.4, 95% CI from 0.2 to 1, *P* = 0.061), although differences were only nearly statistically significant. The distribution of Gorman’s score for diplopia did not differ between ANA-positive and ANA-negative patients (not shown). Similar to CAS, eyelid aperture was lower in ANA-positive patients, with a nearly statistically significant difference (mean difference − 0.9; 95% CI from − 2 to 0, *P* = 0.062).

### Secondary outcome-2: comparison with non-autoimmune hyperthyroidism

Given the relatively high prevalence of ANAs in our patients, we considered the possibility that this could be related to thyroid autoimmunity, to investigate which we compared the prevalence of ANAs in GD patients with that of 78 patients with non-autoimmune hyperthyroidism, namely TNG, who came to our observation over the same period of time, under the same conditions, namely to undergo radioiodine treatment following withdrawal of anti-thyroid medications for 21 days. The main demographic and clinical features of these patients, compared with those of GD patients, regardless of the presence of GO, are reported in Table 3. The two groups differed in terms of: (1) age, which was greater in TNG patients, as expected from epidemiological studies [19]; (2) TSH concentrations, which were lower in TNG patients, as expected from the longer period of anti-thyroid medication withdrawal; and (3) thyroid volume, which was greater in TNG, as also expected from the nature of the two diseases [19]. The remaining features did not differ significantly between GD and TNG.

**Table 3:** Demographic and clinical features of patients with Graves’ disease and patients with toxic nodular goiter (TNG)

	GD (*n* = 265)	TNG (*n* = 78)	Statistics
Gender	Males: 56 (21.1) Females: 209 (78.8)	Males: 16 (20.5) Females: 62 (79.4)	OR: 1 95% CI from 0.5 to 1.9 *P* = 0.906
Age (years)	46.8 (14.1)	61 (13.1)	Mean difference: − 14.1 95% CI from − 17.6 to − 10.6 *P* < 0.0001
Smoking	Never smokers: 138 (52) Ex-smokers: 33 (12.4) Current smokers: 86 (32.4)	Never smokers: 50 (64.1) Ex-smokers: 12 (15.3) Current smokers: 15 (19.2)	Chi^2^: 5.4 *P* = 0.064
Time since diagnosis of hyperthyroidism (months)	24 (12–33.5)	19 (9.7–31)	Mann–Whitney *U*: 2.5 *P* = 0.13
TSH (mU/L) NV: 0.4–4	0.5 (0–1.5)	0 (0–0.2)	Mann–Whitney *U*: 5.9 *P* < 0.0001
FT3 (ng/L) NV: 2.7–5.7	4.4 (3.8–5.5)	4.5 (1–1.4)	Mann–Whitney *U*: 9.3 *P* = 0.27
FT4 (ng/dL) V.N: 0.70–1.70	1.1 (0.9–1.4)	1.2 (0.5)	Mann–Whitney *U*: 9.2 *P* = 0.16
Thyroid volume (mL)	16 (12–22.2)	23.2 (15.5–33.6)	Mann–Whitney *U*: 6.2 *P* < 0.0001

Data are *n* (%), mean (SD) or median (IQR)

*NV* normal values

To our surprise, the proportion of patients with detectable ANAs in TNG was 90%, significantly greater than that in GD patients (Fig. 2b). Similar to patients with GD, also patients with TNG had low ANA titers, namely between 1:80 and 1:160, with no difference between the two groups (not shown). Given the knowledge that ANAs increase with aging [1–4], we postulated that this could explain the difference in prevalence of positive tests between GD and TNG. However, age was not significantly greater in ANA-positive patients, regardless of whether they had GD or TNG (not shown). Likewise, TSH and thyroid volume did not affect the prevalence of ANAs (not shown).

### Secondary outcome-3: ANA patterns

The distribution of ANA patterns in the study population is reported in Table 4. Nuclear staining was in general the one most commonly observed, as reported in the general population [5, 6]. However, there were some differences between GD and TNG ANA-positive patients. Thus, In GD, the nuclear speckled pattern was significantly more frequent, whereas in TNG the nuclear dense fine speckled pattern was nearly significantly more frequent.

**Table 4:** Immunofluorescence patterns of anti-nuclear antibodies (ANA) in patients with Graves’ disease (GD) or toxic nodular goiter

ANA pattern	GD (*n* = 212)	TNG (*n* = 71)	Statistics
Nuclear			
Speckled	29 (13.6)	0 (0)	OR: 22.9, 95% CI from 1.3 to 381.3 *P* = 0.028
Homogenous	31 (14.6)	14 (19.7)	OR: 0.6, 95% CI from 0.3 to 1.4 *P* = 0.31
Dense fine speckled	35 (16.5)	19 (26.7)	OR: 0.5, 95% CI from 0.2 to 1 *P* = 0.059
Fine speckled/fine granular	81 (38.2)	27 (38)	OR: 1, 95% CI from 0.5 to 1.7 *P* = 0.97
Large coarse speckled	11 (5.1)	0 (0)	OR: 8.1, 95% CI from 0.4 to 140.2 *P* = 0.97
Nucleolar	14 (6.6)	5 (7)	OR: 0.9, 95% CI from 0.3 to 2.6 *P* = 0.89
Cytoplasmic	6 (2.8)	3 (7)	OR: 1.2, 95% CI from 0.3 to 5.3 *P* = 0.73
Centromere	1 (0.4)	1 (1.4)	OR: 0.3, 95% CI from 0 to 5.3 *P* = 0.43
Mitotic spindle	4 (1.8)	2 (2.8)	OR: 0.6, 95% CI from 0.1 to 3.7 *P* = 0.63

Data are *n* (%)

## Discussion

The present study, undertaken to investigate a possible relationship between GO and ANAs, stemmed from the lack information on this issue and on somehow conflicting data on the prevalence of detectable ANAs in autoimmune thyroid diseases. To this purpose, we analyzed retrospectively data of a relatively large population (265 consecutive patients) with GD and found a quite impressive prevalence of detectable ANAs, namely 80%. This figure is much greater than the one reported in the general, otherwise healthy population, which is known to be approximately 20% [1–4, 19]. In order to exclude that our observation reflected thyroid autoimmunity, we compared the prevalence of ANAs with that of consecutive patients with non-autoimmune hyperthyroidism, namely TNG, but to our surprise, we found that in this population with no thyroid autoimmunity, the proportion of ANA-positive patients was even greater, namely 90%, with a statistically significant difference with GD. Although in all cases ANAs were detected at relatively low dilution titers (between 1:80 and 1:160), it is quite difficult to explain these unexpected findings. Differences with the healthy populations studied previously [1–4, 20], in terms of age, which is known to affect ANAs [1–4], and possibly other variables, may be responsible for the apparent discrepancy, especially concerning patients with TNG, who are not expected to differ from the healthy population. However, age was not significantly greater in ANA-positive patients, regardless of whether they had GD or TNG. Likewise, TSH and thyroid volume, which were different between GD and TNG patients, did not affect the prevalence of ANAs. We also hypothesized that this unexpected finding could be related to the duration of hyperthyroidism, which, however, did not differ between patients with GD and those with TNG. Another possibility is that hyperthyroidism per se or treatment with anti-thyroid medications, regardless of the presence of autoimmunity, affects ANAs. In this regard, Huang et al. [20] reported a ~ 20% prevalence of ANAs in untreated patients with Graves’ hyperthyroidism, which, however, increased up to ~ 50% upon anti-thyroid treatment. This, coupled with other, still unknown, variables, may be responsible for our findings, to clarify the significance of which further, possibly prospective studies including healthy subjects and considering as many variables as possible, are clearly needed. Whatever the case, comparison between GD and TNG patients seems to indicate a role of thyroid autoimmunity in reducing the prevalence of ANAs or, on the contrary, a protective effect of ANAs in developing thyroid autoimmunity, in this case GD. In this regard, it is worth underscoring that although GD and TNG patients differed for several variables (age, TSH and thyroid volume), none of these affected ANAs, suggesting that the difference was somehow related to the different nature of the two diseases.

The available literature on ANAs in autoimmune thyroid diseases is not conclusive, in that the prevalence of positive tests is quite variable, ranging from 18 to 78% [7, 20–25]. Segni et al. found detectable ANAs in ~ 70% of 93 children with autoimmune thyroid diseases (86 with autoimmune thyroiditis and 7 with GD) [22]. Elnady et al., who studied 61 patients (59 with autoimmune thyroiditis and 2 with GD), including adults (~ 60%), adolescents and children, found a prevalence of detectable ANAs in ~ 70% of them [23]. Nisihara R. et al. [7] reported a prevalence of ~ 20% in 70 patients with autoimmune thyroiditis and 84 with GD. Siriwsrdhane et al. found a ~ 20% prevalence of ANAs in patients with positive tests for anti-thyroid autoantibodies [24]. Paul et al found an impressive ~ 80% ANA prevalence in patients with autoimmune thyroiditis [25]. As mentioned above, Huang et al. [20] reported a ~ 50% prevalence of ANAs in GD. Clearly, to clarify the real prevalence of ANAs in autoimmune thyroid disease, further studies are needed, possibly taking into account a very large number of variables, including ethnicity, age, gender, thyroid function, thyroid treatment and type of thyroid disease.

The primary outcome of the present investigation was the relationship between ANAs and GO. To our knowledge, only one previous study investigated this issue, in a relatively small number of patients, namely 15, and a 20% prevalence of ANAs was found [26]. The frequency of positive tests for ANAs found here in GD patients did not differ between those with or without GO. However, there was a trend to higher ANA titers in patients with GO and, limiting the analysis to patients with GO, we found that proptosis was significantly lower in ANA-positive patients, as were CAS and eyelid aperture, although with only nearly statistically significant differences. The findings seem to suggest a somehow protective role of ANAs on development and severity of GO, which is in line with the observation of a lower prevalence of ANAs in GD compared with TNG, as if ANAs protected not only from GO, but also from developing GD itself and possibly thyroid autoimmunity in general. There were no patients with sight-threatening GO in our cohort, because they are usually referred to thyroid surgery. Further studies are needed to analyze ANAs prevalence, titer and pattern in more severe forms of GO, in comparison with mild and moderate-to-severe GO. Interestingly, there was a different ANA pattern distribution between GD and TNG. Thus, although the nuclear fine speckled/fine granular pattern was the most commonly observed in both groups (~ 40%), the nuclear dense fine speckled pattern, the most common in otherwise healthy subjects [5, 6], was nearly statistically significantly more frequent in TNG, whereas the nuclear speckled pattern was more frequent in GD. The latter pattern is due to the binding of ANAs to proteins constituting the cell pore complexes and is observed in interphase cells [5, 6]. This pattern is associated with mitochondrial antibodies, and it is observed more frequently in patients with primary biliary cirrhosis and other systemic or organ-specific autoimmune diseases, especially of the liver [1–6]. Whether the different pattern distribution is to some extent responsible for the putative protective role of ANAs remains to be established. A possible explanation for our observations is that ANA-positive patients have a switch in T cell population that could protect them from developing GD or could result in a milder clinical GO picture when GO is present. Clearly, this hypothesis is entirely speculative and immunophenotyping analyses should be done to verify whether the presence of ANAs or different ANA patterns correspond to specifically different immune profiles. Therefore, the results of this study are not sufficient to support the role of ANAs in GO, as well as in thyroid autoimmunity and non-autoimmune hyperthyroidism and further studies are needed.

The major limitations of our study are its retrospective design and the absence of a healthy control group, to overcome which prospective, observational, cross-sectional studies are planned.
